# Effect of tranexamic acid on coagulation and fibrinolysis in women with postpartum haemorrhage (WOMAN-ETAC): protocol and statistical analysis plan for a randomized controlled trial

**DOI:** 10.12688/wellcomeopenres.10383.1

**Published:** 2016-12-16

**Authors:** Haleema Shakur, Bukola Fawole, Modupe Kuti, Oladapo Olayemi, Adenike Bello, Olayinka Ogunbode, Taiwo Kotila, Chris O. Aimakhu, Sumaya Huque, Meghann Gregg, Ian Roberts

**Affiliations:** 1 Clinical Trials Unit, London School of Hygiene and Tropical Medicine, London, UK; 2 Department of Obstetrics & Gynaecology National Institute of Maternal and Child Health, College of Medicine, University of Ibadan, Ibadan, Nigeria; 3 Department of Chemical Pathology, College of Medicine, University of Ibadan, Ibadan, Nigeria; 4 Department of Haematology, College of Medicine, University of Ibadan, Ibadan, Nigeria

**Keywords:** Postpartum Haemorrhage, Tranexamic Acid, Fibrinogen, D-dimer, ROTEM

## Abstract

**Background**: Postpartum haemorrhage (PPH) is a leading cause of maternal death. Tranexamic acid has the potential to reduce bleeding and a large randomized controlled trial of its effect on maternal health outcomes in women with PPH (The WOMAN trial) is ongoing. We will examine the effect of tranexamic acid on fibrinolysis and coagulation in a subset of WOMAN trial participants.

**Methods**: Adult women with clinically diagnosed primary PPH after vaginal or caesarean delivery are eligible for inclusion in the WOMAN trial. In a sub-group of trial participants, blood samples will be collected at baseline and 30 minutes after the first dose of tranexamic acid or matching placebo.  Our primary objective is to evaluate the effect of tranexamic acid on fibrinolysis. Fibrinolysis will be assessed by measuring D-dimers and by rotational thromboelastometry (ROTEM). Secondary outcomes are international normalized ratio (INR), prothrombin time (PT), activated partial thromboplastin time (APTT), fibrinogen, haemoglobin and platelets. We aim to include about 180 women from the University College Hospital, Ibadan in Nigeria.

**Discussion:  **This sub-study of WOMAN trial participants should provide information on the mechanism of action of tranexamic acid in women with postpartum haemorrhage. We present the trial protocol and statistical analysis plan. The trial protocol was registered prior to the start of patient recruitment. The statistical analysis plan was completed before un-blinding.

**Trial registration:** The trial was registered: ClinicalTrials.gov, Identifier NCT00872469 https://clinicaltrials.gov/ct2/show/NCT00872469; ISRCTN registry, Identifier ISRCTN76912190 http://www.isrctn.com/ISRCTN76912190 (Registration date: 22/03/2012).

## Background

Postpartum haemorrhage (PPH) is one of the most common obstetric emergencies and is a leading cause of maternal mortality world-wide ^1^. Most of the deaths are in low and middle-income countries and most deaths occur soon after childbirth ^2^. Severe postpartum bleeding can sometimes be managed by the administration of oxytocin and other uterotonic drugs ^3^. However, if uterotonics fail to control the bleeding, surgical intervention may be required.

Tranexamic acid reduces bleeding by inhibiting the enzymatic breakdown of fibrin blood clots by plasmin ^4^. A systematic review of clinical trials of tranexamic acid in surgery showed that it reduces blood loss by about one-third ^5, 6^. Tranexamic acid also reduces mortality in bleeding trauma patients. When given within three hours of injury, tranexamic acid reduces the risk of death due to bleeding by approximately one-third ^7^. Early activation of fibrinolysis is common after trauma and worsens bleeding ^8^. Trauma triggers the release of tissue plasminogen activator (TPA), the enzyme that converts plasminogen to plasmin and the resulting fibrinolysis plays a key role in the pathogenesis of trauma induced coagulopathy. Early administration of tranexamic acid in bleeding trauma patients inhibits fibrinolysis and prevents coagulopathy ^9^.

Increased fibrinolytic activity is also observed after childbirth ^10^. Within 1 hour of delivery, the serum concentration of TPA doubles, possibly due to the trauma of childbirth ^10^. Active PPH is associated with an early increase in D-dimers and plasmin-anti-plasmin complexes ^11^. A randomised trial conducted in obstetric centres in France found that the increase in D-dimers can be inhibited by tranexamic acid administration ^11^. To examine the effect of tranexamic acid on fibrinolysis and coagulation in women at high risk of death after PPH, we aim to conduct a randomised double blind placebo controlled trial in Ibadan, Nigeria (World Maternal Antifibrinolytic Trial-Effect of Tranexamic Acid on Coagulation [WOMAN-ETAC]). 

## Methods

*Hypothesis:* We hypothesise that tranexamic acid will reduce death due to bleeding in women with PPH by inhibiting the breakdown of fibrin clots thus preventing or reducing the severity of coagulopathy. Therefore, our primary aim is to determine the effects of tranexamic acid on indicators of fibrinolysis and our secondary aim is to determine the effect of tranexamic acid on coagulation. 

**Figure 1.**
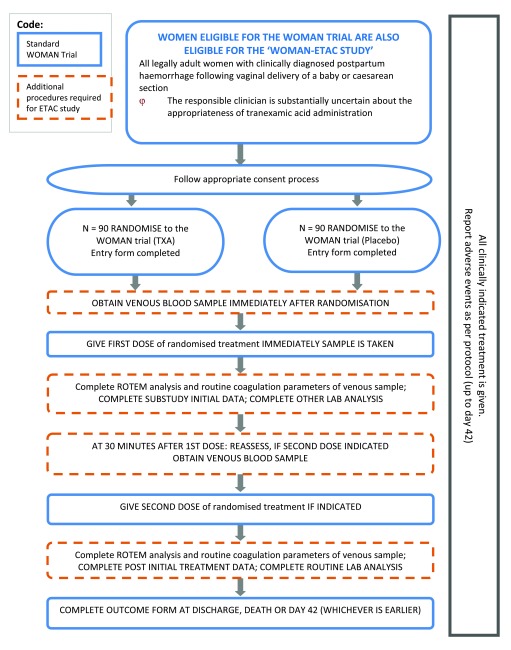
Study overview.

*Design:* A randomised double blind placebo controlled trial will be conducted as a sub-study within the WOMAN trial ( Figure 1). The aims and methods of the WOMAN trial are described in detail elsewhere ^12, 13^. Briefly, adult women with clinically diagnosed primary PPH after vaginal or caesarean delivery are eligible for inclusion. After the appropriate consent procedure has been followed, each patient is randomly allocated to receive 1 gram of tranexamic acid or matching placebo by intravenous injection. If bleeding continues after 30 minutes, or if bleeding stops and restarts within 24 h, a second dose of 1 g of tranexamic acid or placebo may be given. The trial will be conducted in accordance with versions 1 and 1.1 of the protocol.

To examine the effect of tranexamic acid on fibrinolysis and coagulation, in a sample of trial participants we will collect blood at baseline and 30 minutes after the first dose of tranexamic acid or matching placebo. Our primary objective is to evaluate the effect of tranexamic acid on fibrinolysis. Fibrinolysis will be assessed by measuring D-dimers and by rotational thromboelastometry (ROTEM). Secondary outcomes will be international normalized ratio (INR), prothrombin time (PT), activated partial thromboplastin time (APTT), fibrinogen, haemoglobin and platelets. We aim to include about 180 women from the University College Hospital, Ibadan in Nigeria.

*Number of patients needed:* We assume that D-dimer mean and standard deviation in the control group will be 9,000 ng/mL and 7,200 ng/mL respectively. Taking into account that we would adjust for baseline measurement and assuming a correlation between baseline and follow-up of 0.4, we estimate that a study with about 180 patients would have 90% power (two sided alpha=5%) to detect a reduction of 30% in the mean D-dimer value in the tranexamic group. 

*a) Blood sample collection*: Immediately after we randomise a WOMAN trial participant but prior to giving the trial treatment, approximately 15 mL of venous blood will be drawn:

- Two 5 mL samples collected in 5mL vacutainer tubes containing 0.5 mL sodium citrate (0.109mol/L) for coagulation tests and thromboelastometry.
- One 5 mL sample in a 5 mL vacutainer tube containing EDTA.K3 for full blood count analysis.

Once the sample is collected, we will give the first dose of the WOMAN trial treatment according to the WOMAN trial protocol. Approximately 30 (± 15) minutes after administration of the first dose of treatment, we will collect a second blood sample in the same way.

*b) Blood sample analysis:* We will measure D-dimers, INR, PT, APTT and fibrinogen using the HumaClot Junior automated coagulation analyser (Human, GmBH, Germany). We will centrifuge the blood sample at 3000 g for 20 minutes before analysis. We will measure thromboelastometry parameters at 37°C using two of the four channels (EXTEM, APTEM) on the ROTEM coagulation analyser [TEM ^®^, Munich, Germany]). The EXTEM test activates haemostasis by adding tissue factor. The result is influenced by extrinsic coagulation factors, platelets and fibrinogen. EXTEM is a screening test for the (extrinsic) haemostasis system. The APTEM test is an EXTEM based assay in which fibrinolysis is inhibited with aprotinin. A substantial improvement in clot parameters in APTEM compared to EXTEM suggests fibrinolysis. We will obtain a full blood count using a five parameter particle counter Sysmex KN analyser (Sysmex Corporation, Kobe, Japan).

*c) Quality control:* We will store ROTEM reagents in a refrigerator at 2–8°C and will monitor the temperature on a temperature monitoring log. Once opened, ROTEM ^®^ reagents have a limited shelf life (EXTEM, 8 days after opening; APTEM, 14 days after opening). After opening a new bottle of reagent, we will write the expiry date of the reagent on the label. We will not use out of date reagents. We will conduct routine quality control (QC) analyses in accordance with the trial’s standard operating procedures. Only trained personnel will make ROTEM measurements. TEM Innovations GmbH staff will train key personnel before the start of the trial. The lead investigator will train new staff members. We will store ROTEM data on the machine but will take a back-up after each analysis. These will be sent to the Trial Co-ordinating Centre in London. Quality Control of the HumaClot Junior (Human, Germany) will be as per the manufacturer’s instructions . We will file QC reports at the study site so that they are available to the trial team, monitors and auditors.

*d) Other data collection:* We will collect patient entry and outcome data as per the WOMAN trial protocol. In addition, we will collect the following information: time of blood samples, time trial treatment is administered, time laboratory analysis started and ended, any treatment given that may affect coagulation, adverse events, and technical problems with analysis ( Supplementary material). Any untoward medical occurrence affecting a trial participant up to day 42 will be reported in line with the WOMAN trial protocol.

*e) Potential risks to participants:* The study involves two blood tests about 30 minutes apart which may cause pain and bruising at the venepuncture site. The results of the routine laboratory tests can be used to guide treatment in line with local procedures. However, the ROTEM tests will not be used to guide treatment and are for research purposes only.

(f) Roles and responsibilities: The WOMAN-ETAC trial is sponsored by the London School of Hygiene and Tropical Medicine. The Trial Steering Committee (TSC) in place for the WOMAN trial and will be informed of this study. Decisions of the TSC may impact directly the continuation of the WOMAN-ETAC study. If required by the TSC, information about the WOMAN-ETAC study will be reported routinely.

Adverse events which are directly associated with the WOMAN-ETAC study will be reported to the Data Monitoring Committee which is in place for the WOMAN trial. Otherwise there will be no routine review of the accumulating data for the WOMAN-ETAC study.

## Analysis

### Exploratory analyses

We will conduct exploratory analyses to examine the association between clinical parameters (age, type of delivery, blood loss volume, cause of PPH, blood pressure, clinical signs of shock) and the presence or absence of coagulopathy as well as the presence or absence of “hyperfibrinolysis.” Coagulopathy will be defined as an INR >1.2 and A5 ≤ 40mm ^15, 16^. Hyperfibrinolysis will be defined as ML>15% on ROTEM.

We will report the univariate odds ratios for each clinical parameter. All variables will be included in a model to assess multivariate odds ratio. Likelihood ratio tests (LRT) will be used to evaluate statistically if a variable is a risk factor.

### Main analysis

**Figure 2.**
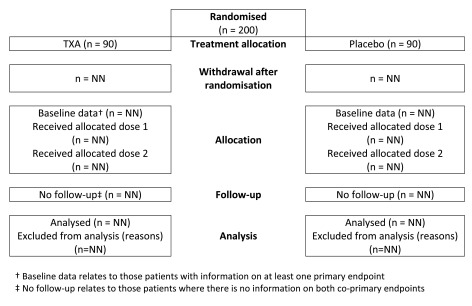
Trial profile.

We will report participant progress through the trial in a Consolidated Standards of Reporting Trials (CONSORT) flow diagram ( Figure 2). We will report the number of participants randomised, allocated to each treatment, lost to follow up or excluded from the analysis (e.g. samples for which there were technical problems with processing). We will conduct per-protocol analysis that include all participants who satisfy the eligibility criteria, receive the allocated treatment, have follow up samples and at least one measurement of the primary outcome. We will not exclude outliers or impute missing data since this would be inappropriate in a study aimed at understanding the biological effects of tranexamic acid. If the coagulation analyser reports, “ *no clot detected*” in lieu of a numeric result, the patient will be excluded from quantitative analysis. However, we will report the number of patients in whom there was no clot detected.

*Baseline data:* We will report participant characteristics and baseline data as numbers and percentages. We will report means and standard deviations for normally distributed parameters and medians and interquartile (25% and 75%) ranges for non-normal distributions. We will test normality using the Shapiro-Wilk test. We will present dichotomous variables as number and percent.

*Co-primary outcomes:* We will assess the effect of tranexamic acid on fibrinolysis by examining d-dimer and maximum clot lysis (ML). D-dimer is a sensitive marker of fibrinolysis. ML (EXTEM ROTEM ^®^) is the percentage reduction after maximum clot formation (maximum strength of the clot firmness reached during the test) at the point of lowest clot amplitude. The time point at which ML is calculated varies, since it is calculated at the point of lowest clot amplitude during the test after maximum clot formation, which varies by participant. EXTEM ML is considered to be one of the most sensitive ROTEM ^®^ measures of fibrinolysis ^15^. If tranexamic acid inhibits fibrinolysis, we would expect to see lower D-dimer measurements and ML percentages in the tranexamic acid group versus placebo. Where the primary outcomes have non-normal distributions, the data will be transformed. We will assume variances to be equal since study participants are drawn from the same population. For each co-primary outcome, we will compare the follow up results of each treatment group (t-test), and conduct a regression analysis that includes the baseline measure.

*Secondary outcomes:* We will assess the effect of tranexamic acid on coagulopathy by examining a range of thromboelastometry parameters using the follow up samples. These will include clotting time (CT), the interval from the start of the test until a clot firmness of 2 mm is reached; clot amplitude at 5 (A5) and ten minutes (A10); maximum clot firmness (MCF) which is the maximum clot amplitude reached during the test. Studies in bleeding trauma patients show that ROTEM A5 is a sensitive indicator of coagulopathy. When coagulopathy is defined as an INR >1.2, a threshold level of EXTEM A5 ≤ 40 mm has a sensitivity of 73% ^16^. If tranexamic acid inhibits fibrinolysis and prevents coagulopathy we would expect higher values for clot amplitude A5, A10, and MCF in the tranexamic acid group compared to placebo. We will also compare the lysis index at 30 (LI30) and 60 minutes (LI60). Lysis index measures the ratio of the firmness of a clot at a given time point and MCF. We expect LI30, LI60 to be higher with tranexamic acid. Additionally, we will assess the difference between follow up results of each treatment group in the following parameters: INR, PT, APTT, fibrinogen and haemoglobin. We will analyse secondary outcomes using t-tests. We will conduct regression analysis that include the baseline measure. 

### Sub-group analyses

*Time since delivery:* In bleeding trauma patients, the effect of tranexamic acid varies according to the interval between injury and treatment. The Clinical Randomisation of an Antifibrinolytic in Significant Haemorrhage (CRASH)-2 trial showed strong evidence that tranexamic acid reduced the risk of bleeding deaths when given within 3 hours of injury but there was no reduction in those treated after 3 hours ^7^. Early fibrinolysis is common after trauma, and is associated with an increase in mortality. Trauma initiates the early release of TPA from storage granules in the vascular endothelium. The release of TPA results in early fibrinolysis that exacerbates bleeding ^8^. Temporal changes in fibrinolysis have also been observed after childbirth. In the first hour after delivery, there is a doubling of the serum concentration of TPA, possibly due to the trauma of childbirth. After the first hour the concentration of TPA decreases rapidly and by a large amount. At the same time, PAI-1 and PAI-2 (plasminogen activator inhibitors) increase around delivery and for several days. To examine temporal changes in fibrinolytic parameters we will report baseline data stratified by time to treatment (≤3 hours after delivery, and >3 hours after delivery). We expect fibrinolysis to be more prevalent in the first three hours. We will also examine the effect of tranexamic acid on fibrinolysis stratified by time to treatment.

*Type of delivery:* Because a substantial proportion of all deliveries are by caesarean section and caesarean section is an established risk factor for PPH, it is important to examine whether the biological effect of tranexamic acid varies by type of delivery. Although we hypothesise that the effects of tranexamic acid on fibrinolysis and coagulation will be similar to those in trauma patients, type of delivery might not accurately reflect the extent to which delivery is traumatic. For example, a study in uncomplicated pregnancy found that D-dimer levels after instrumental vaginal delivery were higher than after spontaneous vaginal birth and similar to those after caesarean section ^17^. We do not anticipate substantial heterogeneity by type of delivery.

*Cause of postpartum haemorrhage:* We will examine the effect of tranexamic acid by cause of bleeding (uterine atony versus all other causes) as determined at baseline. We do not anticipate substantial heterogeneity by cause of haemorrhage.

*Maternal anaemia:* Over fifty million pregnant women are anaemic world-wide ^18^. The highest prevalence of maternal anaemia is in Africa (57%) and in South-East Asia (48%). Anaemia is a risk factor for PPH and venous thromboembolism (VTE) ^19, 20^. Given the high prevalence of anaemia in Africa and Asia, we will examine the effect of tranexamic acid on fibrinolysis and coagulation separately in anaemic and non-anaemic women. We will define anaemia as a haemoglobin concentration less than 110 g/L.

## Declarations

**Ethics approval and consent to participate:** Approvals to conduct this sub-study were obtained from the Ethics Committees of London school of Hygiene and Tropical Medicine (Reference A275 5536) and the University of Ibadan & University College Hospital Ethics Committee (Reference UI/EC/09/0131). Regulatory approval was obtained from the Nigerian National Agency for Food and Drug Administration and Control (NAFDAC). The study will be undertaken according to (International Conference on Harmonisation of Technical Requirements for Registration of Pharmaceuticals for Human Use Good Clinical Practice guidelines) ^21^. The consent procedures will be as detailed in the WOMAN trial protocol ^12^. Briefly, we will seek consent from a patient if their physical and mental capacity allows. If a patient cannot give consent, we will obtain proxy consent from a relative or representative. If a proxy is unavailable, then if local regulation allows, we will defer or waive consent. In this situation, we will inform the patient about the trial as soon as possible, and we will seek consent to use the data. The London School of Hygiene & Tropical Medicine is the sponsor.

**Availability of data and material:** Following publication of the primary and secondary analyses, the trial team will use the data for at least two years for further exploratory analysis. The totally anonymised data will then be made available via our data sharing website: Free Bank of Injury and emergency Research Data (freeBIRD) website ( http://freebird.Lshtm.ac.uk).

## Supplementary material

Data Form
