# Supplementary material for: Effect of tranexamic acid on coagulation and fibrinolysis in women with postpartum haemorrhage (WOMAN-ETAC): protocol and statistical analysis plan for a randomized controlled trial
Source: Wellcome Open Res. 2016 Dec 16;1:31. [Version 1] doi: 10.12688/wellcomeopenres.10383.1 (PMC5351844; doi:10.12688/wellcomeopenres.10383.1)
Supplement: Supplementary file 1 [file wellcomeopenres-1-11189-s0000.tgz › bdd8fabb-e378-4d1f-86cd-070b931e43aa.doc]

**Effect of tranexamic acid on coagulation in a subset of WOMAN trial participants**

**WOMAN–ETAC DATA FORM**

PLEASE ENSURE ALL RELEVANT INFORMATION BELOW IS CONTAINED IN THE MEDICAL RECORDS

| **INITIAL DATA**  PLEASE ENTER ON THE FORM AS PROCESSES ARE COMPLETED | | | | | | | |
| --- | --- | --- | --- | --- | --- | --- | --- |
| **1.** Hospital code *(in your Study File)* |  | | **2.** Patient’s initials *(first name/last name)* | | |  |  |
| **3.** Fully eligible for WOMAN trial? | YES | NO | **4.** Time **baseline** venous blood sample taken *(24-hour clock)* | | | *hours* | *minutes* |
| **5.**Time ROTEM analysis started *(24-hour clock)* | *hours* | *minutes* | ***Note:*** *See overleaf for parameters to be measured – ROTEM data to be printed off and appended to this data form. Data to be transferred to the Coordinating Centre electronically.* | | | | |
| **6.**Routine laboratory samples prepared for analysis *(24-hour clock)* | YES | NO | ***Note:*** *See overleaf for parameters to be measured – copy of data to be printed off, appended to this data form and transferred to the Coordinating Centre.* | | | | |
| **7.** Any treatment which can affect coagulation, given up to 48 hours prior to first blood sample? *(circle one)* | | | YES | NO | *e.g. heparin, warfarin, aspirin, vitamin K, protamine fibrinogen* | | |

| **RANDOMISATION AND FIRST TREATMENT INFORMATION**  PLEASE ENTER ON THE FORM AS PROCESSES ARE COMPLETED | | | | | | | | | | | | | |
| --- | --- | --- | --- | --- | --- | --- | --- | --- | --- | --- | --- | --- | --- |
|  | | | | | | | | | | | | | |
| **8.** Insert randomisation number here | | **BOX** | | | |  |  |  |  | **PACK** | |  |  |
|  | | | | | | | | | | | | | |
| **9.** Date of randomisation   *(24-hour clock)* | *day* | | *month* | *year* | **10.** Time of randomisation  *(24-hour clock)* | | | | | | *hours* | *minutes* | |
| **11.** Time first dose given *(24-hour clock)* | | | | | | | | | | | *hours* | *minutes* | |

| **POST TREATMENT DATA**  PLEASE COMPLETE IMMEDIATELY AFTER 30 MINUTE ASSESSMENT FOR 2^nd^ DOSE,  IMMEDIATELY AFTER 2^nd^ BLOOD SAMPLE AND IF 2^nd^ DOSE IS GIVEN | | | | | | | | | | | |
| --- | --- | --- | --- | --- | --- | --- | --- | --- | --- | --- | --- |
| **12.** Time post-treatment venous blood sample *(24-hour clock)* | *hours* | | *minutes* | **13.** Second dose given? *(circle one)* | | | | | YES | | NO |
| **14.** Time post-treatment ROTEM analysis started *(24-hour clock)* | *hours* | | *minutes* | *Note: See overleaf for parameters to be measured – ROTEM data to be printed off, appended to this data form and transferred to the Coordinating Centre* | | | | | | | |
| **15.** Any treatment which can affect coagulation, given between first and second blood samples? *(circle one)* | | | | | YES | NO | *e.g. heparin, warfarin, aspirin,  vitamin K, protamine fibrinogen* | | | | |
| **16.** Any Adverse Event directly associated with the WOMAN-ETAC study? | | | | | YES | NO | *If YES, report using the WOMAN trial Adverse Event Reporting Procedure* | | | | |
| **17.** Any technical problem with sampling or analysis of venous blood?  *(circle one)* | | | | | YES | NO | *If YES, describe in Box 18* | | | | |
| **18.** | | | | | | | | | | | |
| **19.** a) Name of person completing   this data form | |  | | | b) Date | *day* | | *month* | | *Year* | |
| c) Signature | |  | | | | | | | | | |

**PLEASE ENSURE THE PARAMETERS LISTED BELOW FOR ROTEM AND ROUTINE LABORATORY PARAMETERS ARE COMPLETED FOR BOTH FIRST AND SECOND BLOOD SAMPLES.**

| **ROTEM PARAMETERS TO BE MEASURED** |  | **ROUTINE LABORATORY PARAMETERS  TO BE MEASURED** |
| --- | --- | --- |
| Reaction Time [RT] – Measurement period |  | Haemoglobin |
| Clotting Time [CT] – Time from start to when the waveform  reaches 2mm above baseline |  | White blood cell count |
|  |  | Differential white cell count |
| Clot Formation Time [CFT] – Time from 2mm above baseline  to 20mm above baseline |  | Neutrophils |
|  |  | Lymphocytes |
| Alpha angle [°] α [angle of tangent at 2mm amplitude] |  | Monocytes |
| Maximum angle (CRF) |  | Eosinophils |
| Maximal Clot Firmness [MCF] – Maximum strength |  | Basophils |
| Time to Maximum strength (MCF-t) |  | Platelets |
| Amplitude at a specific time (A5, A10...) |  | Fibrinogen |
| Clot elasticity (MCE) |  | PT |
| Maximum lysis (CLF) |  | INR |
| Clot Lysis[CL] at a specific time [minutes] (LY30, LY45, LY60) |  | APTT ratio |
| Time to lysis (CLT [10% difference from MCF]) |  | D-dimer |

**SENDING DATA TO THE TRIAL COORDINATING CENTRE**

**Data contained on this form:** To be uploaded via web access directly onto the trial database by authorised personnel.

**ROTEM data:** the electronic output should be emailed to [woman.data@Lshtm.ac.uk](mailto:woman.data@lshtm.ac.uk)

**Routine laboratory parameters:** If names are present on the reports, a copy should be made and the name censored on the copy. The randomisation number should be inserted on the form and emailed to [woman.data@Lshtm.ac.uk](mailto:woman.data@lshtm.ac.uk)
