# Supplementary material for: Effect of tranexamic acid on coagulation and fibrinolysis in women with postpartum haemorrhage (WOMAN-ETAC): protocol and statistical analysis plan for a randomized controlled trial
Source: Wellcome Open Res. 2016 Dec 16;1:31. [Version 1] doi: 10.12688/wellcomeopenres.10383.1 (PMC5351844; doi:10.12688/wellcomeopenres.10383.1)
Supplement: Peer review history

# Peer review: Referee response for version 1

**DOI:** 10.21956/wellcomeopenres.11189.r21335

**Published:** 2017-03-29

**Authors:** Anne-Sophie Ducloy-Bouthors, Emmanuelle Jeanpierre, Sophie Susen

**Affiliations:** 1 Pôle d'Anesthésie-Réanimation, Hôpital Jeanne-de-Flandre, CHRU Lille, Lille, France; 2 Centre de Biologie Pathologie, Centre Hospitalier Régional, Lille, France; 3 Institut Pasteur de Lille, Faculté de Médecine - Pôle recherche, University of Lille Nord de France, Lille, France

This article describe the WOMAN-ETAC protocol as a substudy of the WOMAN trial.

 In 180 women from the University College Hospital, Ibadan in Nigeria, fibrinolysis will be assessed at baseline and 30 minutes after the first dose of tranexamic acid or matching placebo, by measuring D-dimers and by rotational thromboelastometry (ROTEM). Secondary outcomes are international normalized ratio (INR), prothrombin time (PT), activated partial thromboplastin time (APTT), fibrinogen, haemoglobin and platelets.

 The article is correctly written and the ethics, quality and administrative research controls are well configured.

 Some questions and suggestions may help to improve the manuscript.

**Recruitment**:

- Please specify if the 180 patients are the consecutive series of the Nigerian center, allowing a blinded unselected distribution of the groups among the substudy.

P4: **Number needed to study**:

- The term "about" should be avoided. If the objective of 180 is not reached, then explain. In Figure 2 trial profile, n=200?
- The control group expected level of D-dimers is 9000±7200 ng/mL.
- Please clarify the timing of this expected control level in the course of the bleeding. How was this high level estimated?

When considering the D-dimers levels in untreated and treated haemorrhagic patients in Exadeli trial, the values were as follow (Ducloy-Bouthors *et al*. 2016), slightly less than your control group level.

         Group Control untreated Haemorrhagic n=72                                                          

                    Enrolment      +30 min       +2 h         +6 h      *P value        

 D-dimers                            

 (ng ml−1)        3730            6158          7495          4936       0.0001             

                 [2468–8493]  [3600–10000]  [4400–15772]  [2905–8278]                  

         TA treated Haemorrhagic n=72

                   Enrolment      +30 min        +2 h            +6 h        *P value

 D-dimers                            

 (ng ml−1)       3645              5556         3888           2687       0.004

                [2222–6223]  [3087–7598]   [2751–6123]††  [1768–4502]††

 This could modify the number needed to observe the TA effect on D Dimers.

 P4 and 5: **ROTEM parameters.to diagnose hyperfibrinolysis:**

- Please clarify the type of ROTEM analyzer: delta or sigma.
- Concerning APTEM test, please make sure with TEM-international that the antifibrinolytic reagant used has been changed from aprotinin to tranexamic acid and precise the concentration of the APTEM reagant.
- Please clarify if the quality control Rotrol will be performed by the trained personnel every week as commonly or before each of test.
- Replace "is administered" by "administration"
- Lysis onset time LOT has been used to increase the performance of ROTEM analysis to detect and diagnose hyperfibrinolysis (Faraoni *et al. 2015*) should be also an interesting parameter to be tested

p5: **Definition of the coagulopathy**:

- The definition of the coagulopathy seems to be based on the trauma definition of the coagulopathy and hyperfibrinolysis: INR >1.2 and A5 < 40mm and ML>15% on ROTEM respectively.
- The PPH associated coagulopathy and ROTEM cutoff have been described by Huissoud (2009) then Collins (2014).
- Because the thrombin generation and plasmin generation seems very close and appears both early in the course of PPH (Ducloy-bouthors *et al. *P-011&-012, 2017), and the decrease in fibrinogen seems parallel to the increase in D-dimers, the cutoffs established by Collins to diagnose PPH induced coagulopathy should be used. Because ISTH DIC score is not sensible in PPH population and the new score has been validated (Jonard *et al.* 2016), the threshold to should be adapted

P6: **Secondary parameters:**

- Timed measure of fibrinolytic activity after childbirth needs a better description in non haemorrhagic as well as untreated and treated haemorhagic groups. The WOMAN-ETAC data will be very helpful to this better understanding and ongoing TRACES trial () will complete these data. The duration of both the hyperfibrinolytic activity and the tranexamic action is also very useful to establish with no previous data.
- The duration and intensity of the antifibrinolytic activity should be interconnected with the estimated bleeding volume and the initial estimated bleeding flow. To be aware of the estimated initial bleeding volume and flow, two methods may be used and mutually consolidated: the visual estimation (although imperfect) and the hemoglobin delta between end of pregnancy and the first available hemoglobin (if possible before dilution due to vascular loading). The resuscitation and transfusion parameters should be considered to understand the variation of the coagulopathic and fibrinolytic process.
- Type of delivery: Delivery is not spontaneously traumatic and obstetrics coagulation activation should not be considered as a trauma induced coagulation and fibrinolysis activation. The aim of this coagulation and fibrinolysis activation is the physiological closure of the placental vascular bed after placental removal. This coagulation and fibrinolytic activation remains local and uterine-limited since the uterine tone blocks the dissemination of the large amounts of tissue factor. In trauma patients, initial shock and tissue damage are directly correlated to coagulopathy and mortality, whereas initial obstetrics coagulation and fibrinolysis activity is rarely associated with shock, even in late and severe evolution when transfusion and vascular loading are given.
  Thus as the authors expect, the type of delivery will not influence when considering the placental bed coagulation and fibrinolytic activation. However in the instrumental delivery as well as in caesarean section, the surgical part of the bleeding may be so severe that the hypovolemic shock could be associated. Perhaps should it be possible to analyze in clinical parameters the origin of bleeding and to confirm these data by a lactate measurement on the initial and 30 minutes blood samples.
- Maternal anemia is an interesting topic where tranexamic acid may reach an important public health objective. Goswani *et al. *(2013) already showed this favourable impact in prophylactic use during non haemorrhagic CS.

We have read this submission. We believe that we have an appropriate level of expertise to confirm that it is of an acceptable scientific standard.

## References

[1] Ducloy-BouthorsASDuhamelAKipnisETournoysAPrado-DupontAElkalioubieAJeanpierreEDebizeGPeynaud-DebayleEDeProstDHuissoudCRauchASusenS: Postpartum haemorrhage related early increase in D-dimers is inhibited by tranexamic acid: haemostasis parameters of a randomized controlled open labelled trial. *Br J Anaesth*.2016;116(5) : 10.1093/bja/aew021 641-8 10.1093/bja/aew021 27106967

[2] FaraoniDVan der LindenPDucloy-BouthorsASGoobieSMDiNardoJANielsenVG: Quantification of Fibrinolysis Using Velocity Curves Measured with Thromboelastometry in Children with Congenital Heart Disease. *Anesth Analg*.2015;121(2) : 10.1213/ANE.0000000000000795 486-91 10.1213/ANE.0000000000000795 26049780

[3] HuissoudCCarrabinNAudibertFLevratAMassignonDBerlandMRudigozRC: Bedside assessment of fibrinogen level in postpartum haemorrhage by thrombelastometry. *BJOG*.2009;116(8) : 10.1111/j.1471-0528.2009.02187.x 1097-102 10.1111/j.1471-0528.2009.02187.x 19459866

[4] CollinsPWLilleyGBruynseelsDLaurentDBCannings-JohnRPreciousEHamlynVSandersJAlikhanRRaymentRReesAKayeAHallJEParanjothySWeeksACollisRE: Fibrin-based clot formation as an early and rapid biomarker for progression of postpartum hemorrhage: a prospective study. *Blood*.2014;124(11) : 10.1182/blood-2014-04-567891 1727-36 10.1182/blood-2014-04-567891 25024304

[5] GoswamiUSarangiSGuptaSBabbarS: Comparative evaluation of two doses of tranexamic acid used prophylactically in anemic parturients for lower segment cesarean section: A double-blind randomized case control prospective trial. *Saudi J Anaesth*.2013;7(4) : 10.4103/1658-354X.121077 427-31 10.4103/1658-354X.121077 24348295PMC3858694

[6] Ducloy-BouthorA.S.JeanpierreEHennartBBaptisteA.S.GiovannoniSSaidiISimonELannoyDAllorgeDDuhamelASusenS: Thrombin and plasmin generation as measured by a single well technique during haemorrhagic and non-hemorrhagic caesarean section: pilot study. *Thrombosis Research*.2017;151(1) : 10.1016/S0049-3848(17)30110-X S113 10.1016/S0049-3848(17)30110-X

[7] Ducloy-BouthorsA.S.JeanpierreEHennartBGiovannoniSSaidiISimonELannoyDAllorgeDDuhamelASusenS: TRAnexamic acid to reduce blood loss in hemorrhagic CESarean delivery: therapeutic and pharmaco-biological dose-ranging multicenter randomized double blind placebo controlled study: TRACES trial methodology. *Thrombosis Research*.2017;151(1) : 10.1016/S0049-3848(17)30109-3 S113 10.1016/S0049-3848(17)30109-3

[8] JonardMDucloy-BouthorsASFourrierF: Comparison of Two Diagnostic Scores of Disseminated Intravascular Coagulation in Pregnant Women Admitted to the ICU. *PLoS One*.2016;11(11) : 10.1371/journal.pone.0166471 e0166471 10.1371/journal.pone.0166471 27861597PMC5115738

# Peer review: Referee response for version 1

**DOI:** 10.21956/wellcomeopenres.11189.r20530

**Published:** 2017-03-06

**Authors:** Andrew D. Weeks

**Affiliations:** 1 Sanyu Research Unit, Department of Women's and Children's Health, Liverpool Women's Hospital, University of Liverpool, Liverpool, UK

This is a well thought-out study and will add important information to the literature. Given that the study has now finished, and the data analysis plan finalised and the study un-blinded (as per the abstract discussion) it would be inappropriate to suggest any changes to the protocol or analysis. There are however a number of ways in which the protocol could be written more clearly so as to ensure clarity for those wishing to replicate or understand the study:

1. In METHODS (b) it would be good to state exactly how long after the blood sample was taken it was analysed and whether any steps were taken to ensure that the sample did not deteriorate (eg kept on ice, or analysed within a certain time period)
2. In the same section it would be good to be explicit about how long the ROTEM protocol lasted and stating that (I assume) the ROTEM was allowed to run for the full X minutes. This is important for the assessment of lysis as that may continue to change for some time and so lysis data would be different if the ROTEM was interrupted at 10 minutes or at 20 minutes.
3. Can the authors clarify who took the samples and put them through the ROTEM analysis, and whether the results were then revealed to the clinicians? If it was, it could have the effect of unblinding those doing the clinical care and collecting the data.
4. In ANALYSIS, the definition of ML and A5 need to be clarified.
5. I assume that there are 2 of each of the ROTEM measurements for each participant with one relating to the EXTEM and the other for the APTEM. If not, then please clarify this in the script – but if there is, then each description of MCF, A5 etc should clarify which measure it comes from.
6. At the bottom of ‘Main analysis’ it states that the machine may not give data if there was 'no clot detected'. Is this correct? Surely the sodium citrate will prevent clot formation and it is only if ‘Clot Detected’ that the analyser will not work.

I have read this submission. I believe that I have an appropriate level of expertise to confirm that it is of an acceptable scientific standard.

# Peer review: Referee response for version 1

**DOI:** 10.21956/wellcomeopenres.11189.r19830

**Published:** 2017-02-01

**Authors:** Pollyanna Hardy

**Affiliations:** 1 National Perinatal Epidemiology Unit, University of Oxford, Oxford, UK

***Title and Abstract***: The title and abstract are appropriate for the content of the article.

***Article content: ***The study design and methods are explained clearly and with sufficient detail. I have focussed on the analysis section as this is where my expertise lies.

 Some consideration should be given to the possibility of under-representation of women with more severe PPH, and how that may affect the conclusions of the study. Since some time is required to take the blood samples before the first dose of study drug is administered, is there a possibility that the more severe cases of PPH will not be included in this sub-study, or may have a baseline value but no follow-up value? An analysis examining the representativeness of the women included in the sub-study versus the remainder in the main WOMAN study may help to assess this issue.

 The use of a per-protocol analysis population is reasonable, given the objective of the study. With regard to the definition of this population, should this also exclude women who have received a second dose of study drug before the follow-up blood samples were taken, in the rare cases when this might happen? Have the authors considered conducting a sensitivity analysis using an intention to treat analysis population to assess the robustness of the conclusions?

 The authors specify that they will use t-tests as well as regression analyses adjusting for baseline values to analyse the outcomes. They should make clear which analysis method will be considered the primary analysis. It would also be helpful to include how the outcome data will be reported (for example as adjusted mean differences), and if confidence intervals and/or p-values will be reported. The level of confidence intervals for the primary and secondary outcome results should also be specified, as well as the level to be used for statistical significance.

 The rationales for each of the sub-group analyses are clearly described. A brief description of the method of analysis should also be included. Will tests of interaction be reported?

 There is a minor error in the numbers reported in Figure 2. The number randomised should be 180, not 200.

I have read this submission. I believe that I have an appropriate level of expertise to confirm that it is of an acceptable scientific standard.
